# Characterization of the β-defensin genes in giant panda

**DOI:** 10.1038/s41598-018-29898-2

**Published:** 2018-08-17

**Authors:** Zhi-Yi Zhang, He-Min Zhang, De-Sheng Li, Tie-Yi Xiong, Sheng-Guo Fang

**Affiliations:** 1 The Key Laboratory of Conservation Biology for Endangered Wildlife of the Ministry of Education, and State Conservation Centre for Gene Resources of Endangered Wildlife, College of Life Sciences, Zhejiang University, Hangzhou, 310058 P. R. China; 2 China Research and Conservation Center for the Giant Panda, Wolong, 623000 P. R. China; 3 Emeishan Biological Resources Research Station, Sichuan Provincial Academy of Natural Resources Sciences, Chengdu, 610015 P. R. China

## Abstract

β-Defensins are small antimicrobial proteins expressed in various organisms and have great potential for improving animal health and selective breeding programs. Giant pandas have a distinctive lineage in Carnivora, and it is unclear whether β-defensin genes have experienced different selective pressures during giant panda evolution. We therefore characterized the giant panda (*Ailuropoda melanoleuca*) β-defensin gene family through gap filling, TBLASTN, and HMM searches. Among 36 β-defensins identified, gastrointestinal disease may induce the expression of the *DEFB1* and *DEFB139* genes in the digestive system. Moreover, for *DEFB139*, a significant positive selection different from that of its homologs was revealed through branch model comparisons. A Pro-to-Arg mutation in the giant panda DEFB139 mature peptide may have enhanced the peptide’s antimicrobial potency by increasing its stability, isoelectric point, surface charge and surface hydrophobicity, and by stabilizing its second β-sheet. Broth microdilution tests showed that the increase in net charge caused by the Pro-to-Arg mutation has enhanced the peptide’s potency against *Staphylococcus aureus*, although the increase was minor. We expect that additional gene function and expression studies of the giant panda *DEFB139* gene could improve the existing conservation strategies for the giant panda.

## Introduction

Defensins are a group of small antimicrobial peptides (AMPs) found in large variety of plants, invertebrates, and vertebrates that act against a broad spectrum of pathogens including enveloped viruses, gram-positive and gram-negative bacteria, mycobacteria, and fungi^1^. There are three defensin subfamilies known as α, β, and θ defensins; among these, β-defensins represent the oldest family, and most mammalian β-defensins arose before the last common ancestor of eutherian mammals^1–4^. β-defensin proteins are expressed mainly by mucosal epithelial cells lining the respiratory, gastrointestinal, and genitourinary tracts, but the expression pattern and antimicrobial properties of many β-defensins remain unknown^1,4^.

Apart from killing microbes directly, recent studies have discovered novel roles for β-defensins in immunology and reproduction. Navid *et al*. (2012) found that mouse β-defensin 14 (Defb14) can mediate the immune response by regulating T cells^5^. Human DEFB2, DEFB3, and their mouse orthologs are also chemoattractants used for recruiting immune cells to fight against infection^6^. Hardwick *et al*. (2011) found higher *DEFB103* expression levels in the epithelial cells of people from East Asia^7^, which can increase influenza resistance in this population. In macaques, spermatozoa coated with DEFB126 are protected from attack by the female immune system, which facilitates fertilization^8,9^. Furthermore, certain variants of some β-defensins in human and cattle are also related to fertility^8,10^. Because of these properties, several potential applications for β-defensins can be envisioned for improving animal health, including the design of antibiotics that would avoid the emergence of resistance, the development of vaccine adjuvants, dietary manipulations that enhances gut disease resistance, and selective breeding for breeds with higher fitness^1,11,12^.

Giant pandas represent the ‘national treasure’ of China and a flagship for world wildlife conservation. These animals have received much attention not only because of their endangered status, but also because of their intriguing evolutionary history. To date, many genetic analyses have investigated the phylogeny, genetic variation, population history, adaptive evolution, and structure of this species using a combination of molecular markers including fingerprint probes, microsatellites, mitochondrial DNA, and major histocompatibility complex (MHC) loci^13^. As one of the most important disease resistance genes, MHCs in the giant panda are extensively studied to assess their immunological adaptation and fitness, which ultimately provides guidance for captive breeding and reintroduction programs^14–16^.

Despite the promising applications of β-defensins in animal health, research on these immune genes in the giant panda has been largely overlooked. The number of β-defensin gene loci in various databases is currently large but many have an incomplete gene structure, and it is therefore difficult to target suitable genes for further study. With the advent of genome sequencing, the development of bioinformatics tools, and our giant panda BAC library^17^, we were able to depict the full repertoire of this subfamily. Furthermore, we aimed to identify any giant panda β-defensin genes that have adaptively evolved to cope with changing pathogens, as a dietary switch from meat to bamboo may have generated a microflora distinct to the giant panda^18^.

## Results

### Characterization of the giant panda β-defensin genes

**Figure 1:**
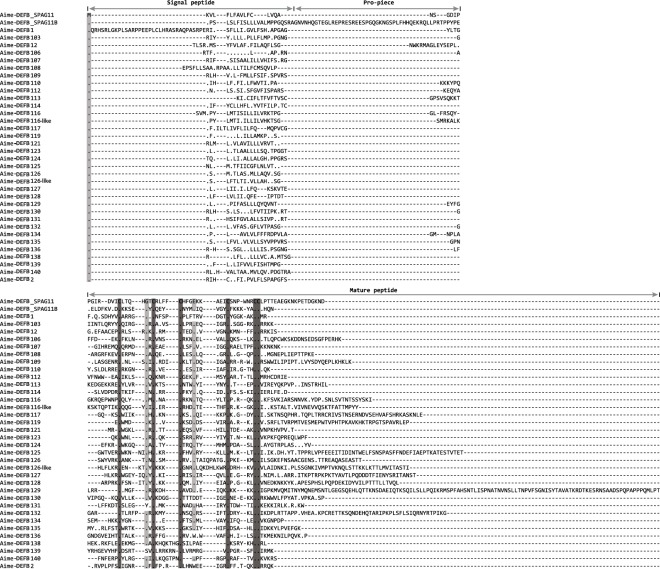
Amino acid sequence alignment of β-defensin proteins in giant panda. The name of the β-defensins starts with the abbreviation of the species’ Latin name followed by the name of the β-defensin. Dots and dashes represent identity and alignment gaps compared to Aime-DEFB_SPAG11, respectively. Regions corresponding to the signal peptide, pro-piece, and mature peptide domains are indicated. Light grey: conserved residues (frequency ≥70%); dark grey: six highly conserved cysteine motifs.

Thirty-six β-defensins with intact structures were identified in the giant panda genome (Fig. 1 and Supplementary Table S1). As in other mammals, most β-defensin genes in giant panda have two exons that encode a signal peptide, a short pro-piece, and a mature peptide domain^1^. One exception is the sperm-associated antigen 11B (SPAG11B); in different mammals, this peptide has three exons with a long pro-piece encoded by the second exon. It is unique that DEFB1 in the giant panda (Aime-DEFB1) has a long signal peptide. Although the signal peptide domain in Aime-DEFB1 could not be detected using SignalP, the 36^th^ to 56^th^ residues of the peptide resembled the structure of a signal peptide (Fig. 1). An alignment of the sequence with DEFB1 in the polar bear showed that the elongated Aime-DEFB1 signal peptide might result from a Met-to-Arg mutation at the 33^rd^ residue. The expression of the *DEFB1* gene was also detected in our study, providing evidence of a functional *DEFB1* gene in the giant panda (Table 1).

**Table 1:** Expression patterns of four β-defensin genes in the giant panda. A plus symbol (+) indicates the expression of the β-defensin in the indicated tissue.

Tissue	Aime-*DEFB1*	Aime-*DEFB103*	Aime-*DEFB139*	Aime-*DEFB140*
Blood		+		
Liver		+		
Ovary	+	+		
Spleen	+		+	
Kidney	+	+	+	
Stomach	+		+	
Pancreas	+			+

**Figure 2:**
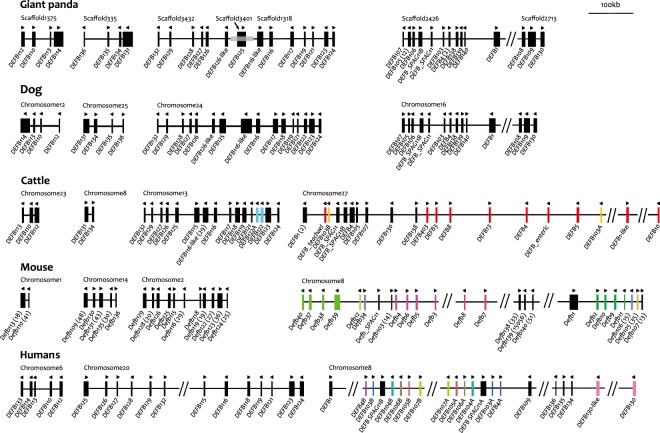
Syntenic relationships between β-defensins in the giant panda (*A*. *melanoleuca*), dog (*Canis lupus familiaris*), cattle (*Bos Taurus*), mouse (*Mus musculus*), and humans (*Homo sapiens*). Vertical bars represent defensin genes and their orientation is indicated by a black triangle. The location of each gene, cluster, and the BAC clone 1054F4 (grey shade) is annotated and their length is drawn to scale (upper right corner). When a gap between two neighbouring genes was larger than 100 kb, it is noted as “//”. Paralogs are depicted with the same colour, and orthologs are noted with the same name based on the results of phylogenetic analyses (Supplementary Fig. S1). The number in the bracket indicates the original number assigned by NCBI.

These 36 β-defensin genes are distributed on seven scaffolds: scaffold 1375, 335, 3432, 3401, 1318, 2426 and 2713 (accession numbers GL192431.1, GL193056.1, GL194707.1, GL195409.1, GL192588.1, GL194420.1, and GL195214.1, respectively). As the BAC clone (1054F4) covered scaffold 3401 and also bridged scaffolds 3432 and 1318, a total of five giant panda β-defensin gene clusters were observed (Fig. 2). According to our orthological analysis, these five gene clusters should be distributed on four different chromosomes (Fig. 2). The β-defensin genes in the giant panda had the strongest synteny with those in the dog, as almost all of the β-defensin genes in these two species showed one-to-one orthological conservation (Fig. 2), in contrast to the many gene duplications that occurred in humans, mice and cattle (Fig. 2).

**Figure 3:**
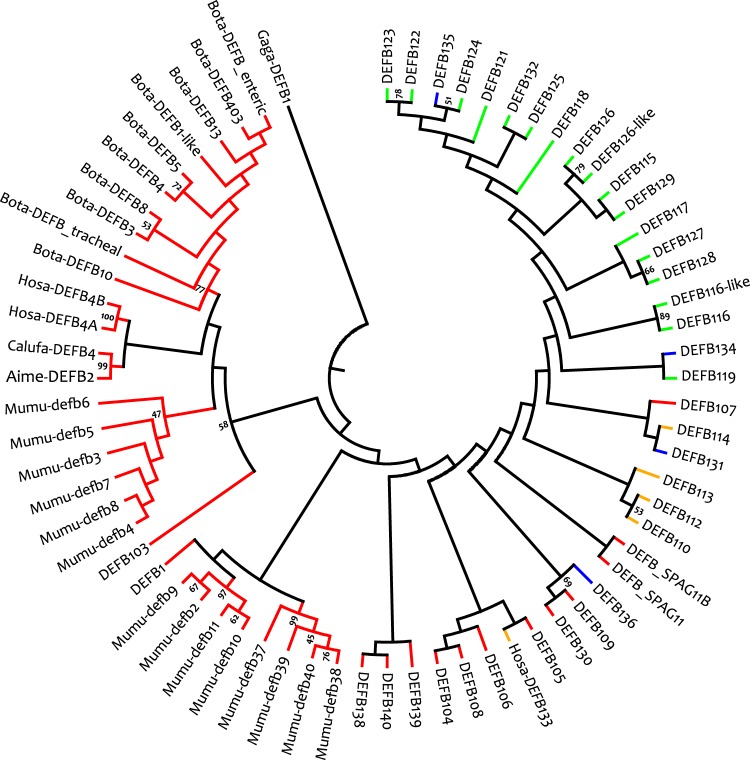
Neighbour-joining tree of β-defensins in the giant panda, dog, cattle, mouse, and humans. The chicken (*Gallus gallus*) β-defensin 1 (i.e. Gaga-DEFB1) is used as an outgroup and bootstrap values under 40 are not shown. Genes conserved as one-to-one orthologs or which only have two copies in some species are collapsed into one branch, whereas branches with duplicated genes in cattle or mice remain expanded. On expanded branches, the name of the genes starts with the abbreviation of the species’ Latin name followed by the β-defensin name. For detailed evolutionary relationships for each gene, please refer to Supplementary Fig. S1. Lineages from the same chromosome are depicted in the same colour.

When the reconstructed phylogeny of β-defensins in humans, mice, cattle, dogs, and giant pandas was evaluated, only *DEFB110*, *DEFB113*, *DEFB116*, *DEFB119*, *DEFB123*, *DEFB124*, *DEFB125*, *DEFB129*, and *DEFB131* dispayed a one-to-one orthology (Fig. 3). These genes mostly belonged to one β-defensin gene cluster that was evolutionarily the youngest of the clusters identified^12^. Other genes either had duplications or were lost in some species. For example, *DEFB104*, *DEFB115*, *DEFB118*, and *DEFB122* were not represented in the giant panda genome, whereas in mice, cattle, and humans there were many gene duplications in the most ancient β-defensin gene cluster^12^ (Fig. 3 and Supplementary Fig. S1). Specifically, several duplications of *DEFB4* were present in cattle, whereas the mouse *Defb4* was duplicated and then diverged during evolution (Fig. 3). There were also several mouse-specific gene duplications, including *Defb37-40* and *Defb9-10* (Fig. 3). In humans, *DEFB4*, *DEFB130* and *DEFB103-107* exist as two copies in the genome. Figure 3 also shows that some neighbouring defensins such as *DEFB4* and *DEFB103*, *DEFB116* and *DEFB116-*like, and *DEFB126* and *DEFB126-*like clustered with high bootstrap support values, indicating that *cis* duplication was common during the evolution of immune gene families^19^.

### Expression pattern of β-defensins in the giant panda

We only detected the expression of four β-defensin genes in the various tissues investigated in the giant panda used in this study. In the giant panda, *DEFB1*, *DEFB103*, and *DEFB139* were expressed in more tissues than *DEFB140* (Table 1). Because the health status of the studied individual and sample size could affect the results of β-defensin gene expression profiling, we BLAST-searched these gene sequences against several giant panda tissue transcriptome datasets. Although no transcriptome data were found for the spleen, kidney and pancreas, we still observed a broad spectrum of expression of the *DEFB1* and *DEFB103* genes; *DEFB1* was expressed in the liver (accession number SRX1208869), ovary (SRX1208780), and pituitary (SRX1208761), while *DEFB103* was expressed in the blood (SRX542925), skin (SRX1208682), and tongue (SRX1208772). The expression of these four genes was not found in the transcriptome data corresponding to the digestive tract. However, the expression of *DEFB1* and *DEFB139* in the digestive system observed in our study could have been due to acute haemorrhagic enteritis present in the specific giant panda used in the study.

### Evolutionary analyses of the expressed giant panda defensin genes

**Figure 4:**
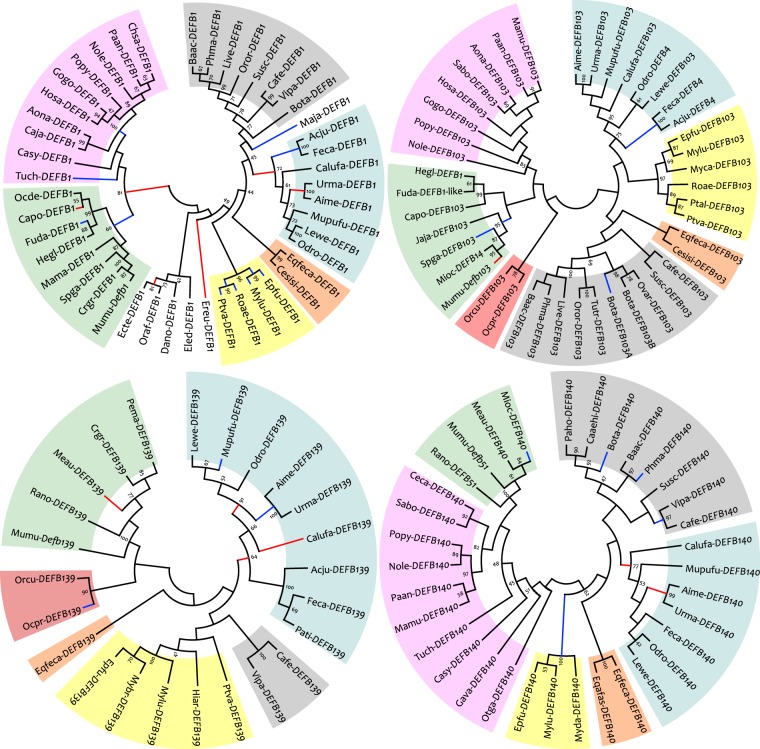
Maximum likelihood trees of the *DEFB1*, *DEFB103*, *DEFB139*, and *DEFB140* genes. Purple, green, red, yellow, orange, blue, and grey indicate Euarchonta, Rodentia, Lagomorpha, Chiroptera, Perissodactyla, Carnivora, and Cetartiodactyla, respectively. Support values under 40 are hidden. Branches under episodic positive selection (empirical Bayesian factor, EBF > 60), irrespective of whether the mature peptide region or entire sequence were used in the analyses, are shown in blue. Branches in red indicate lineages with EBF > 60 only when the entire sequence was analysed. The Latin species names and their abbreviations are as follows: *Acinonyx jubatus* (Acju), *A*. *melanoleuca* (Aime), *Aotus nancymaae* (Aona), *Balaenoptera acutorostrata* (Baac), *B*. *taurus* (Bota), *Callithrix jacchus* (Caja), *Camelus ferus* (Cafe), *C*. *lupus familiaris* (Calufa), *Capra aegagrus hircus* (Caaehi), *Carlito syrichta* (Casv), *Cavia porcellus* (Capo), *Cebus capucinus* (Ceca), *Ceratotherium simum simum* (Cesisi), *Chlorocebus sabaeus* (Chsa), *Cricetulus griseus* (Crgr), *Dasypus novemcinctus* (Dano), *Echinops telfairi* (Ecte), *Elephantulus edwardii* (Eled), *Eptesicus fuscus* (Epfu), *Equus africanus asinus* (Eqafas), *Equus ferus caballus* (Eqfeca), *Erinaceus europaeus* (Ereu), *F*. *catus* (Feca), *Fukomys damarensis* (Fuda), *Galeopterus variegatus* (Gava), *Gorilla gorilla* (Gogo), *Heterocephalus glaber* (Hegl), *Hipposideros armiger* (Hiar), *H*. *sapiens* (Hosa), *Jaculus jaculus* (Jaja), *Leptonychotes weddellii* (Lewe), *Lipotes vexillifer* (Live), *Macaca mulatta* (Mamu), *Manis javanica* (Maja), *Marmota marmota* (Mama), *Mesocricetus auratus* (Meau), *Microcebus murinus* (Mimu), *Microtus ochrogaster* (Mioc), *M*. *musculus* (Mumu), *Mustela putorius furo* (Mupu), *Myotis brandtii* (Mybr), *Myotis capaccinii* (Myca), *Myotis davidii* (Myda), *Myotis lucifugus* (Mylu), *Nomascus leucogenys* (Nole), *Ochotona princeps* (Ocpr), *Octodon degus* (Ocde), *Odobenus rosmarus* (Odro), *Orcinus orca* (Oror), *Orycteropus afer* (Oraf), *Oryctolagus cuniculus* (Orcu), *Otolemur garnettii* (Otga), *Ovis aries* (Ovar), *Panthera tigris* (Pati), *Pantholops hodgsonii* (Paho), *Papio anubis* (Paan), *Peromyscus maniculatus* (Pema), *Physeter macrocephalus* (Phma), *Pongo pygmaeus* (Popy), *Propithecus coquereli* (Prco), *Pteropus alecto* (Ptal), *Pteropus vampyrus* (Ptva), *Rattus norvegicus* (Rano), *Rhinolophus sinicus* (Rhsi), *Rousettus aegyptiacus* (Roae), *Saimiri boliviensis* (Sabo), *Spalax galili* (Spga), *Sus scrofa* (Susc), *Tupaia chinensis* (Tuch), *Tursiops truncatus* (Tutr), *Ursus maritimus* (Urma), *Vicugna pacos* (Vipa).

This represents the first study to identify giant panda β-defensins important for defending against gastrointestinal disease, which is common in giant pandas. We next further analysed these four genes using phylogeny and identified their different evolutionary history. The Z-test showed that for each gene, the mean dS was larger than the mean dN, suggesting a significant purifying selection. However, at the whole-sequence level, several sites were under episodic positive selection, and the BUSTED analysis indicated episodic positive selection for *DEFB1*, *DEFB103*, and *DEFB139* (Table 2). Episodic positive selection on *DEFB1* was most frequent in several taxa, whereas for *DEFB139*, periodic positive selection appeared to be confined to genes in carnivores (Fig. 4).

**Table 2:** Results of the Z-test for selection and BUSTED analyses.

Gene	Z-test of selection		BUSTED
	Test^1^/Test^2^	p^1^/p^2^	p^1^/p^2^
*DEFB1*	3.42/3.28	0.000/0.001	0.004/0.011
*DEFB103*	6.02/6.46	0.000/0.000	0.006/0.020
*DEFB139*	3.55/2.64	0.000/0.005	0.021/0.064
*DEFB140*	4.35/4.04	0.000/0.000	0.600/0.756

The “Test” column shows the result of dS-dN, and p < 0.05 indicates significant negative selection. P-values in the BUSTED column smaller than 0.05 suggest episodic positive selection on the gene. Superscripts 1 and 2 indicate the analyses that were used for the entire sequence or mature peptide domain, respectively.

**Figure 5:**
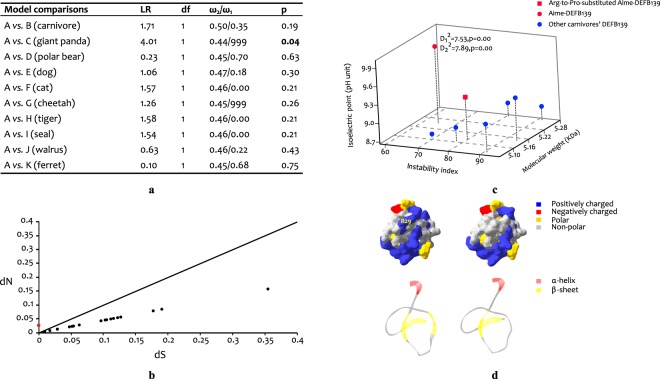
Branch model comparisons of the *DEFB139* gene and changes in the Aime-DEFB139 mature peptide induced by the point mutation. (**a**) Branch model comparisons using the entire coding sequence. Model A is a one-ratio model. Models B to K are two-ratio models. The tested branches are shown in the brackets. ω1 and ω2 are ω values for tested and reference lineages, respectively. LR and df represent the differences of the two models in likelihood ratio and degree of freedom, respectively. Only the two-ratio model of Aime-*DEFB139* had a significantly better fit than the null model in describing the selective pressures on *DEFB139* orthologs. (**b**) Scatter plot of dN/dS values from model C for each species. The red dot is Aime-*DEFB139* and the reference line is represented by dots with dN/dS = 1. (**c**) Physiochemical properties of the DEFB139 mature peptide in different carnivores. Original and manually adjusted (i.e. replacement of the 29^th^ Arg to Pro) Aime-DEFB139 variants are represented by filled red circles and squares, respectively. The remaining carnivore DEFB139 homologs are represented by filled blue circles. D_2_^2^ and D_1_^2^ are the Mahalanobis distances calculated with and without the Arg-to-Pro-substituted Aime-DEFB139, respectively, and the critical values calculated for these datasets according to Eq. (1) are 7.46 (df1 = 3, df2 = 10, α = 0.01) and 6.76 (df1 = 3, df2 = 9, α = 0.01), respectively. Therefore, only the Aime-DEFB139 mature peptide was found to be an outlier with regard to physiochemical properties, including the instability index and isoelectric point. Exact measurements of the physiochemical properties and Mahalanobis distances for each DEFB139 mature peptide are provided in Supplementary Table S4. (**d**) Tertiary structure model comparisons. Models on the left and right depict the molecular surface and tertiary structure of the mature peptide for the original and Arg-to-Pro-substituted Aime-DEFB139, respectively. Different residues and secondary structures are indicated by different colours.

When only the mature peptide domain was analysed, a significant episodic positive selection was no longer detectable for *DEFB139* (Table 2), and relatively fewer branches (i.e. three out of seven) of *DEFB139* remained under episodic positive selection (Fig. 4). This suggests that nonsynonymous substitutions in the signal peptide played an important part in the evolution of the *DEFB139* gene; thus, we analysed the selective pressure on the signal peptide region of *DEFB139* (Supplementary Methods). Our results showed that the DEFB139 signal peptides in non-carnivores were under significant purifying selection (dS-dN = 2.54, p = 0.01), whereas the purifying selection for this signal peptide in Carnivora was reduced (dS-dN = 1.55, p = 0.06). Moreover, the signal peptide region in Aime-*DEFB139* (the giant panda *DEFB139*) diverged the most from that of non-carnivores (Supplementary Table S2). Therefore, the complete Aime-*DEFB139* coding sequence was evaluated first using branch models to reflect the selective pressure on this gene. We found that among the several two-ratio models tested, only the two-ratio model that tested the ω (dN/dS) value in the giant panda lineage out-competed the null one-ratio model (Fig. 5a,b). The significantly higher dN/dS rate in the giant panda lineage suggested a different evolutionary history from that in other mammalian lineages; however, this rate was not significant when either the mature peptide (dN/dS = 999, p = 0.09) or signal peptide domain (dN/dS = 999, p = 0.13) of Aime-*DEFB139* gene was analysed separately. This indicates that the unique evolutionary history of *DEFB139* in the giant panda contributed to the nonsynonymous substitutions throughout the whole sequence rather than in a single domain.

Because the branch model only compares averaged ω values for whole gene sequences, we applied RELAX, the Branch-Site Model, and MEME, which account for the selective pressure on specific sites, to examine changes in selective pressure and to recognize sites where selection has occurred. Contrary to the branch model analyses, RELAX only suggested a near-significant positive selection on the entire Aime-*DEFB139* coding sequence, but this selective pressure was the most strengthened (Supplementary Table S3). Furthermore, when the whole coding sequence was examined, the Branch-Site Model (Bayes Empirical Bayes, BEB = 0.93) and MEME (p = 0.14) indicated that the 29^th^ residue of the Aime-DEFB139 mature peptide was most likely subjected to diversifying selection (the two methods have critical significance levels of BEB > 0.95 and p < 0.10, respectively).

### Physiochemical properties and structure of the giant panda DEFB139 mature peptide

The 29^th^ residue is an Arg and only presents in the giant panda DEFB139 and large brown bat DEFB139; in all other mammals tested, a Pro is retained at this position. This Pro-to-Arg mutation is likely to represent a monomorphism (Supplementary Note), and by acquiring this additional positively charged residue, the Aime-DEFB139 mature peptide displays reduced instability and a higher isoelectric point (Supplementary Table S4). According to the instability index, Aime-DEFB139 is still unstable; however, proteins such as RNase A and β-defensins are stabilized in their native forms by several disulfide bridges^20^. Such structures are known to make proteins resistant to degradation, which results in a misleading instability index^20,21^. Therefore, the Aime-DEFB139 mature peptide may or may not be unstable following the Arg mutation. Moreover, similar to the Aime-DEFB139 mature peptide, an Arg rather than a Pro between Met and Gly residues could make proteins more resistant to degradation^20^. Similarly, the increased isoelectric point alone cannot guarantee a higher antimicrobial potency, but the isoelectric point and antimicrobial potency of β-defensins are positively related^22^. In terms of the isoelectric point and instability index, the Aime-DEFB139 mature peptide differs significantly (p = 0.00) from its orthologs according to the calculated Mahalanobis distances (D^2^; Fig. 5c), which indicated an increased antimicrobial activity for this protein in the giant panda. Moreover, our modelling results suggested that the Aime-DEFB139 mature peptide displays a different molecular surface, and its second β-sheet had appeared after the point mutation had occurred (Fig. 5d). The increased surface charge and hydrophobicity, as well as its stabilized second β-sheet, may also be related to the enhanced antimicrobial potency of Aime-DEFB139^22,23^.

### Antibacterial activity of the giant panda DEFB139

To examine the influence of the Pro-to-Arg mutation on the antimicrobial activity of the Aime-DEFB139 mature peptide, we attempted to synthesize high purity Aime-DEFB139 and Arg-to-Pro-substituted Aime-DEFB139 (i.e. the Aime-DEFB139 with its Arg residue replaced by Pro) with the native folding pattern, but failed. This is because β-defensins contain six Cys residues, which can be easily oxidized and lead to isoforms with distinctive disulfide pairings^24^. Moreover, the additional positively charged Arg in Aime-DEFB139 could have induced an electrostatic repulsion that prevented the required hydrophobic packing during the *in vitro* chemical synthesis; and the Cys potentially could not get close enough to form disulfide bonds^24^, which resulted in partially oxidized variants that also lowered the product’s purity.

Considering that the antimicrobial activity of β-defensins against some bacteria is independent of the presence and pattern of disulfide bridges^21,22^, we synthesized the two peptides with their Cys residues protected by acetamidomethyl (Acm). Although these peptides will maintain their linear form, as Acm prohibits the formation of disulfide bridges, this modification will not change the peptides’ net charge, a main factor (rather than disulfide bonding) that affects the antimicrobial potency of β-defensins^21,22^. Therefore, we can still evaluate the effect of the increased net charge in Aime-DEFB139 due to the Pro-to-Arg mutation. Furthermore, as the peptides were kept in their linear form, isoforms and partially oxidized variants did not occur, which has led to high purity products. The synthesis of the Acm-protected Aime-DEFB139 produced a 97.8% pure product with a mass of 5608 Da (the theoretical value is 5,607.2 Da, and this difference was within the error range^25^; Supplementary Fig. S2). The Acm-protected Aime-DEFB139 in which the Arg-to-Pro substitution was engineered was obtained with 97.3% purity and with a mass of 5,548.9 Da (theoretically, the mass is 5,548.1 Da; Supplementary Fig. S2).

The CD spectra of the two synthesized peptides are very similar (Supplementary Fig. S3), and their secondary structure only differs slightly, as calculated using the CDPro packages. Both peptides contain almost the same fractions of α-helix (about 7%) and β-turn (about 25.3%), while Aime-DEFB139 is a little less unordered and exhibits a slightly higher proportion of β-sheet than the Aime-DEFB139 containing the Arg-to-Pro substitution (26.1% and 23.6%, respectively).

**Figure 6:**
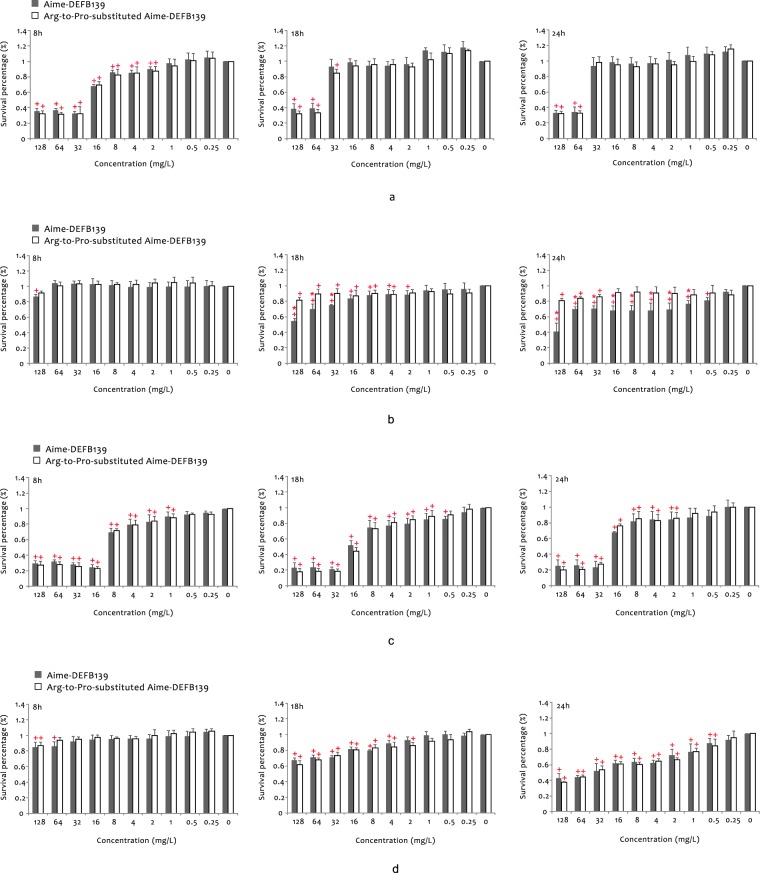
Antimicrobial activities of the Acm-protected Aime-DEFB139 and Arg-to-Pro-substituted Aime-DEFB139 against (**a**) *E*. *coli*, (**b**) *S*. *aureus*, (**c**) *K*. *pneumoniae* and (**d**) *Y*. *enterocolitica* at different time points. The diluted strains were cultivated with different β-defensin concentrations (0–128 mg/L) for 24 h. At different time points, the optical density was measured at 600 nm. The turbidity ratio in the wells containing each β-defensin to those without the β-defensin peptides was calculated as the survival percentage. A plus symbol (+) indicates that a particular β-defensin at a certain concentration had a statistically significant antimicrobial activity (detailed p-values are in Supplementary Table S5). An asterisk (*) indicates that at a certain concentration, Aime-DEFB139 had a statistically significantly (P < 0.05) higher antimicrobial potency than the Arg-to-Pro-substituted Aime-DEFB139 (detailed p-values are shown in Supplementary Table S6).

The two synthesized peptides suppressed bacterial growth at low concentrations. However, to kill substantial loads of *Staphylococcus aureus*, *Escherichia coli* and *Yersinia enterocolitica* (>50%), they still had to reach a high concentration (above 30 mg/L) which may be non-physiological (Fig. 6a–d). Although in statistical terms the antimicrobial potency of Aime-DEFB139 against *S*. *aureus* was significantly higher than that of the Arg-to-Pro-substituted Aime-DEFB139 (Fig. 6b), this enhancement in potency was physiologically minor.

## Discussion

Our study initially analysed the annotation of the β-defensin gene family in the giant panda genome and the evolutionary relationships between these genes and other mammalian β-defensin genes. The gene family has undergone gain and loss evolutionary process which are typical for gene families involved in immune function^26^. Although the *DEFB104*, *DEFB115*, *DEFB118*, and *DEFB122* genes in the giant panda have been lost (Supplementary Fig. S1), this is not unique, as further BLAST searches showed that carnivores such as dogs, cats, polar bears, and ferrets have also lost some of these genes over their evolutionary history. Because conserved β-defensins may not provide enough information for a clear and robust resolution between orthologs, a poorly supported orthologous relationship between some defensins in the giant panda and the dog (e.g. *DEFB140*, *DEFB110*, and *DEFB107*) was observed (Supplementary Fig. S1). Nevertheless, the *DEFB140*, *DEFB110*, and *DEFB107* genes in the giant panda are most similar to their orthologs in the polar bear based on the results of our BLAST searches. Therefore, the conserved synteny and well-supported orthology suggests that the giant panda genome still retains the typical carnivore β-defensin gene family. This was expected because, similar to many other immune gene families, the purifying selection on the β-defensin gene family resulted in structural and functional conservation^26^.

Generally, although carnivore β-defensin sequences are conserved, we detected a significant positive selection on the Aime-*DEFB139* gene through branch model comparisons (Fig. 5). However, the significant increase in selective pressure on the Aime-*DEFB139* gene should be attributed not only to the nonsynonymous substitutions in the mature peptide, but also to those in the signal peptide domain. The reduced purifying selection on the signal peptide may be related to changes in secretion efficiency or expression specificity^27^. We hypothesize that the mutation at the 29^th^ residue in the Aime-DEFB139 mature peptide may be related to an increase of antimicrobial potency.

We speculate that the point mutation identified in the Aime-DEFB139 mature peptide is pathogen-driven and crucial for enhancing the peptide’s antimicrobial potency. First, the residue is in the mature peptide where the molecule interacts with the pathogens^28^. Second, the Pro-to-Arg substitution made the Aime-DEFB139 mature peptide unique by enhancing its isoelectric point and stability (Fig. 5c,d). Several studies have suggested that antimicrobial potency can be positively correlated with the cationic charge and stability of β-defensins;^21,23,24,29–31^ therefore, Aime-DEFB139 has a distinctive antimicrobial activity when compared to its orthologs in other carnivores. Third, in DEFB139 homologs present in other carnivores, replacing the 29^th^ residue of the mature peptide with an Arg does not alter its secondary structure. However, this is not the case for Aime-DEFB139, where a second β-sheet appears in the peptide after the replacement of Pro by Arg (Fig. 5d). CD spectroscopy also suggested that the Pro-to-Arg mutation has changed the β-sheet content of the Aime-DEFB139. Therefore, this indicates that the Arg residue could be important for the stability of the β-sheet in Aime-DEFB139. If the Aime-DEFB139 mature peptide dimerizes through the second β-sheet similar to the human β-defensin 3, which is the product of the human *DEFB103* gene, when attacking microbes^22^, this very site could be critical for the function of Aime-DEFB139. Lastly, its relatively broad spectrum of expression (Table 1), and especially its expression in the stomach, where diverse and new pathogens are confronted by the host system, may have promoted the high antimicrobial potency of Aime-DEFB139.

Branch-site model-based analyses did not identify any positive selection on Aime-DEFB139. This is probably because the positive selection may have been masked by the strong purifying selection. If nonsynonymous substitutions are exclusive to Aime-DEFB139, the substitution could be regarded as a chance appearance rather than being caused by positive selection, even if the latter is true^32^. For example, the 29^th^ residue of the DEFB139 mature peptide was only mutated in the giant panda and large brown bat, while remaining conserved in all other mammals; indicating that the positive selection at this site is thus masked by the purifying selection.

Branch-site based analyses also require sufficient proportions of nonsynonymous substitutions caused by significant positive selection for the results of a tested lineage to be statistically significant^33–35^. The significant change in β-defensin genes in some organisms is due to distinctive transformations in their lifestyle and/or living environment. For example, β-defensins in cattle and mice have duplicated under positive selection as they adapted to their specialized niches, such as rumen development and inhabiting environments with complex microbes, respectively^4,12,36^. The expanded number of *AvBD1* and *AvBD3* genes in songbirds such as the zebra finch and hwamei is thought to be linked with structures required to maintain their singing trait, such as a highly developed syrinx^37^. Furthermore, as the ocean has a microbial environment very different from that of terrestrial habitats, the selective pressure on defensins, such as *DEFB103* and *DEFB140*, in cetaceans has been significantly relaxed (Supplementary Table S7 and Fig. S4), which has ultimately led to the disappearance of these genes (Fig. 4). In comparison, although the giant panda has adopted a bamboo-dominant diet, the change in its intestinal microbiota appears trivial^38^; thus, there may not have been a substantial change in the living environment to generate a strong selective pressure on the β-defensin gene family. Even if a special gut microbiome has evolved in giant pandas, the change may only have occurred approximately four million years ago (MYA)^39^, whereas the mammalian β-defensin gene family was established more than 100 MYA^4,9,40^. This indicates that changes in microbial composition may not provide a sufficiently long selective pressure to overwhelmingly alter the β-defensins in the giant panda. Thus, the positive selection was not strong and long enough for giant panda defensins to accumulate substantial substitutions under dominant purifying selection.

Indeed, there may be no need for the giant panda to accumulate many nonsynonymous substitutions in its β-defensins for these to be different from their homologs in other carnivores. In some immune peptides, including β-defensins, a single amino acid substitution is sufficient to alter their molecular potency against pathogens^30^. Targeting a broad range of pathogens, Aime-DEFB139 may not need to change significantly under diversifying selection to cope with new pathogens, and an enhanced antimicrobial activity resulting from a point mutation could be sufficient for this coping ability. To show that the Pro-to-Arg mutation in Aime-DEFB139 has changed the peptide’s antimicrobial potency, we synthesized the Aime-DEFB139 mature peptide and the Arg-to-Pro-substituted Aime-DEFB139 with their Cys residues protected by Acm, and then compared their antimicrobial activity against *E*. *coli*, *S*. *aureus*, *Y*. *enterocolitica*, and *K*. *pneumoniae* using the broth microdilution assay. Our results suggested that the increase in net charge caused by the Pro-to-Arg mutation indeed can enhance the peptide’s potency against certain bacteria, although the enhancement was minor.

The minor difference in antimicrobial potency between the two synthesized peptides and their low efficacy in killing bacteria may be due to the lack of disulfide bonding, since only after correct folding and dimerization can some β-defensins exhibit enhanced antimicrobial potency^21,41,42^. Compared with the Pro-containing Aime-DEFB139 peptide, a correctly folded Aime-DEFB139 peptide could have a much higher antimicrobial potency, as it has a higher net charge and more stable β-sheets, which may enable its dimerization. Another explanation for the minor difference in antimicrobial potency between the two peptides could be that rather than affecting antimicrobial activities, the Pro-to-Arg mutation may mainly influence the functions that require a higher order structure (e.g. chemotactic activities)^24^, as the mutation affects the stability of the peptide’s secondary structure. The best but also the most labour-intensive, time consuming, and costly way to resolve the above questions regarding the functions of the Aime-DEFB139 peptide would be to selectively form disulfide bonds to ensure its native folding pattern^22^.

In conclusion, *DEFB139* is likely to represent a special β-defensin gene in the giant panda, and its expression and function are worth further study to enhance the health and fertility of giant pandas. As the dietary supplementation of butyrate enhances disease resistance by up-regulating β-defensins in the gut^11,43^, it is useful to evaluate whether a dietary supplementation could also stimulate the expression of the Aime-*DEFB139* gene in the digestive tract and thus improve the health of giant pandas in captivity, who tend to lack butyrate-producing bacteria^38^. Another gene expressed in the blood, Aime-*DEFB103*, is also worth mentioning because it provides a non-destructive approach to the study of the adaptive evolution of giant pandas. The use of blood samples could help identify changes in Aime-*DEFB103* gene expression in different giant panda populations. Identification of a link between the gene expression and fitness in a population would aid in the selection of suitable individuals for breeding. Furthermore, the scope and depth of future studies could also be improved by including samples from different individuals, as our study focused on genes expressed in samples taken from one individual panda that died from acute haemorrhagic enteritis, which could have affected the expression of defensin genes. Because the acquisition of tissue samples from giant pandas is challenging, increased collaboration between different institutions will be needed in the future to conduct more comprehensive studies in this species.

## Methods

### Ethics statement

All the samples used in this study were collected with permission from the China Research and Conservation Center for the Giant Panda and the State Forestry Administration. The blood sample was collected from the arm vein of a male individual bred in the China Research and Conservation Center for the Giant Panda, and was obtained by the technical staff of the centre with utmost care. The centre also provided other preserved tissue samples, including the heart, kidney, liver, ovary, pancreas, spleen, stomach, and small and large intestines that were collected from an individual that had died of acute haemorrhagic enteritis. All experiments were approved by the ethics committees of the China Research and Conservation Center for the Giant Panda and were performed in accordance with the approved guidelines.

### Search for new defensin genes in giant pandas

To identify potential novel β-defensins in the giant panda, initial TBLASTN searches were conducted on the nucleotide collection (nr/nt), reference genomic sequences (refseq_genomic), high-throughput genomics sequences (HTGS), and whole genome shotgun contigs (WGS) databases of the giant panda available at the National Center for Biotechnology Information (NCBI). These searches used all existing β-defensins in the giant panda together with orthologs exclusive to humans (*Homo sapiens*), mice (*Mus musculus*), cattle (*Bos taurus*), and dogs (*Canis lupus familiaris*) as query sequences. Hits with an E-value < 0.1 were examined manually for the typical motif or conserved signal sequence of β-defensin. For each potential newly identified gene, additional iterative searches were performed using the above method until no more sequences could be discovered. All searches used the default settings of the NCBI web site tools.

A hidden Markov model (HMM) analysis was used as a complementary strategy to search for novel β-defensins. Previously identified amino acid β-defensin sequences in the giant panda were aligned using ClustalW in MEGA 6.0 and trimmed to retain the core of the mature peptide where the conserved six-cysteine motif is located^44^. The alignment was then submitted to the hmmsearch program on the HMMER web server to construct a HMM^45^, which was then used to query the UniProt Knowledgebase (UniProtKB) using default settings. Potential hits were checked for the six-cysteine motif and additional iterative searches were performed as described above. Mammalian β-defensins have a signal sequence and mature peptide separated by an intron. Therefore, when a motif lacked a signal section or vice versa, a flanking sequence of 5–20 kb was retrieved to derive the full length and structural organisation of the gene by using a combination of SignalP^46^, GENSCAN^47^, Fgenesh^48^, and GeneWise^49^.

Because new defensin genes tend to arise through duplication and form clusters, the sequences between two neighbouring defensins were also retrieved and scanned using Genescan and Fgenesh to find distant homology. Moreover, assuming that closely related genes (bootstrap value above 55), including *DEFB103* and *DEFB2*, *DEFB109* and *DEFB130*, *DEFB116* and *DEFB116*-like, *DEFB126* and *DEFB126*-like, and *DEFB128* and *DEFB129*, could have other recent duplicates nearby, the gaps around these genes would be filled through PCR if larger than 120 bp because the mature β-defensin peptide has at least 40 amino acids. When a gap was too wide to be filled by PCR, we applied BAC walking based on our lab’s panda BAC library to cover the gap^17^, and then used the identified clones as a template for amplification using universal primers for the flanking defensin genes. PCR products, if any, were sequenced and scanned using a combination of Genescan and Fgenesh to check for novel β-defensins. The primers used are listed in Supplementary Tables S8 and S9.

### Evolutionary analyses

A phylogenetic tree of β-defensins in the giant panda, dog, cattle, mouse, and humans was reconstructed using MEGA 6.0. The program was employed to align the translated mRNA sequences (Supplementary Data S1 and S2) and then select the best substitution model to generate a neighbour-joining tree using the chicken DEFB1 as an outgroup. One thousand bootstrap replicates were performed to assess node support. Maximum likelihood trees were also constructed for both nucleotide and amino acid sequences using PhyML^50^. As there was no significant difference in the clustering of the orthologs, these trees are not presented. In addition, the relative positions and orientations of all β-defensins from different species were investigated using BLAT^51^.

The expression of *DEFB1*, *DEFB103*, *DEFB139*, and *DEFB140* was detected in the giant panda; therefore, these four genes were chosen for further phylogenetic analyses. To better reflect their phylogeny, we included their orthologs, which were retrieved from NCBI (all accession numbers and sequences of the species included are in Supplementary Data S3 and S4). MEGA 6.0 was used to build phylogenetic trees with a maximum likelihood algorithm. Nucleotide sequences were aligned, and the best nucleotide substitution model was chosen for each alignment. A fast subtree-pruning-regrafting search using a weak branch swap filter was enforced on an initial maximum parsimony tree. One thousand bootstrap replicates were performed to assess node support.

For each gene, the overall mean rates of nonsynonymous (dN) and synonymous (dS) substitutions in the entire peptide and mature peptide domain, and the significance of the overall selection, were evaluated by Z-test of selection in MEGA 6.0 using a modified Nei and Gojobori method with the Jukes-Cantor correction, 1,000 bootstrap replicates, and a 90% site coverage cut-off. The overall selective pressure on the entire sequence and mature peptide domain were also measured using BUSTED on the datamonkey web server, which identified gene-wide evidence of episodic positive selection based on sites under periodic positive selection^33,52^.

To examine the hypothesis that particular defensins in the giant panda have evolved under positive selection to handle changes in pathogenic exposure, we built branch models for the whole peptide and separately for the mature peptide region using the CodeML program in PAMLX to evaluate the dN/dS (ω) variation between different carnivore lineages and the rest of the tree^53^. In each analysis, the null one-ratio model, which assigns the same ω to all branches, was compared with the alternative two-ratio model. The latter model estimates one ω for a single carnivore lineage treated as a foreground branch (i.e. tested lineage) and assigns another ω for the remaining branches (i.e. reference lineages). If the alternative model where the panda branch has a different ω is the best fit, we can infer that the panda β-defensin was under an evolutionary scheme different from that of other mammals. This analysis was followed by applying a Branch-Site Model in CodeML and MEME on the datamonkey web server to identify specific sites under positive selection^32^. MEME can also identify branches under periodic positive selection. RELAX, on the datamonkey web server, was used as a complementary analysis to determine whether selective pressure on the panda β-defensins had intensified during evolution^35^ (detailed in Supplementary Methods).

### Expression pattern of β-defensins in the giant panda

Total RNA from tissues isolated from the giant panda was extracted using phenol-chloroform and Trizol according to the manufacturer’s instructions (Invitrogen, Carlsbad, CA). After the evaluation of RNA quality by agarose gel electrophoresis, cDNA was synthesized from 2–3 μg of RNA using the TAKARA Synthesis kit (Takara, Shiga, Japan, PrimeScript RT reagent Kit with gDNA Eraser) following the manufacturer’s instructions. Primers spanning the intron were designed (Supplementary Table S10) for each giant panda β-defensin gene, and the expression patterns of these defensin genes were examined in each tissue.

### Analysis of physiochemical properties and modelling of the Aime-DEFB139 mature peptide

The physiochemical parameters closely related to antimicrobial activities of AMPs, including isoelectric point and stability were calculated with the Isoelectric Point Calculator and ProtParam^22,29–31,54,55^, respectively. To examine whether β-defensins from giant panda have distinctive physiochemical characteristics that may indicate a different antimicrobial activity from that of other carnivore β-defensins, the differences within the orthologs were tested using the Mahalanobis distance calculated using EXCEL 2007 (Microsoft Co., Ltd.), which is a popular method for detecting outliers in multivariate data^56^. The Mahalanobis distance represents the distance from an observation to its prediction, where the observations are assumed to have a Gaussian distribution in the null hypothesis. If the null hypothesis is rejected, we can conclude that outliers from the Gaussian distribution exist. According to Penny (1996), the critical value for a small sample should be calculated as:

1 $$\frac{d{f}_{1}\times {(d{f}_{2}-1)}^{2}\times {F}_{d{f}_{1},d{f}_{2}-d{f}_{1}-1;\alpha /d{f}_{2}}}{d{f}_{2}\times (d{f}_{2}-d{f}_{1}-1+d{f}_{1}\times {F}_{d{f}_{1},d{f}_{2}-d{f}_{1}-1;\alpha /d{f}_{2}})}$$

where df_1_ is the number of parameters measured, df_2_ refers to the number of observations for each parameter, and the significance level α is divided by df_2_ to take multiple statistical tests into account (i.e. Bonferroni bounds)^56^.

The tertiary structure of β-defensin proteins in the giant panda was also predicted. The giant panda sequences were subjected to the SWISS-MODEL to identify the most similar sequence to provide the basis for modelling the peptides’ tertiary structure^57^. The models were further analysed using Swiss-PdbViewer v4.10^58^.

### Antibacterial activity assay

To compare the antibacterial activities of the giant panda DEFB139 mature peptide and its respective Aime-DEFB139 peptide in which an Arg-to-Pro substitution was engineered, we chemically synthesized the two peptides. The two peptides, YRHGEVYHFC (Acm) DSRTSVC (Acm) LRRKRNC (Acm) LVRMRGVC (Acm) PGRSFC(Acm) C (Acm) IRMK (i.e. Aime-DEFB139) and YRHGEVYHFC (Acm) DSRTSVC (Acm) LRRKRNC (Acm) LVRM***P***GVC (Acm) PGRSFC (Acm) C (Acm) IRMK (i.e. Arg-to-Pro-substituted Aime-DEFB139), were synthesized through solid-phase peptide synthesis by the Chinese Peptide Company (Hangzhou, China). The same company purified all peptides by HPLC and verified their molecular weight by electrospray ionization mass spectrometric (ESI-MS). The detailed information of syntheses, purification, and characterizations were reserved by the company.

The two peptides were analyzed by CD spectroscopy using the Jasco J-1500 Circular Dichroism Spectrometer (JASCO, Inc., Easton, MD). The samples were prepared at 200 mg/L in sterile deionized water directly before measurement. CD measurements were performed at room temperature within a 180–280 nm wavelength range. Every sample was scanned three times. The smoothed and averaged data were generated by the instrument’s accompanying software and then analysed by using the CDPro software package^59^.

The broth microdilution method described by Wiegand *et al*.^60^ was used to evaluate the antimicrobial activities of the two antimicrobial peptides against *E*. *coli* ATCC25922, *S*. *aureus* ATCC25923, *Y*. *enterocolitica* CMCC52204, and *K*. *pneumoniae* ATCC 13883, which are pathogenic bacteria found in the intestinal tract of giant pandas. Each strain was diluted to approximately 5 × 10^5^ CFU/mL in quarter-strength Mueller-Hinton broth (MH broth), and each peptide was prepared at 2560 mg/L in sterile deionized water. The individual peptides were then diluted in a continuous concentration gradient to 256, 128, 64, 32, 16, 8, 4, 2, 1, and 0.5 mg/L with 100 μL of quarter-strength MH broth in a 96-well cell culture plate (Costar 3599, Corning, Inc., Corning, NY). A 100 μL growth control containing no peptide and a 200-μL sterility control without inoculum were also included. Then, 100 μL of the diluted strain was mixed with 100 μL of the individual peptides at different concentrations, such that the final concentrations of the peptide in each well were 0–128 mg/L. The plates were incubated at 37 °C, and the turbidity at 600 nm at 8 h, 18 h and 24 h was measured using a microplate reader (SpectraMax 190, Molecular Devices, San Jose, CA). The survival percentage of bacteria was defined as the ratio of the turbidity in the wells with the peptide to that without the peptide. All experiments were performed in triplicate on two independent occasions. All values are expressed as the mean + standard deviation (SD).

We used Minitab 17 (Minitab Inc.) to conduct an analysis of variance (ANOVA) to determine whether a peptide has antimicrobial activity at different concentrations. Means were compared using Tukey’s and Dunnett’s tests. Significant differences in antimicrobial activity between the two peptides at a specific concentration were determined using the Student’s t-test. Statistical significance was defined at p < 0.05.

### Data availability

All data generated or analyzed during this study are included in this published article and its Supplementary Information files.

## Electronic supplementary material

Supplementary information

Dataset 1

Dataset 2

Dataset 3

Dataset 4
