# Supplementary material for: Characterization of the β-defensin genes in giant panda
Source: Sci Rep. 2018 Aug 17;8:12308. doi: 10.1038/s41598-018-29898-2 (PMC6098084; doi:10.1038/s41598-018-29898-2)
Supplement: Supplementary file 1 — Supplementary information [file 41598_2018_29898_MOESM1_ESM.pdf]

Characterization of the β-defensin genes in giant panda

Zhi-Yi Zhang[1], He-Min Zhang[2], De-Sheng Li[2], Tie-Yi Xiong[3] and Sheng-Guo Fang[1]*

[1] The Key Laboratory of Conservation Biology for Endangered Wildlife of the Ministry of Education, and State Conservation Centre for Gene Resources of Endangered Wildlife, College of Life Sciences, Zhejiang University, Hangzhou 310058, P. R. China.

[2] China Research and Conservation Center for the Giant Panda, Wolong 623000, P. R. China.

[3] Emeishan Biological Resources Research Station, Sichuan Provincial Academy of Natural Resources Sciences, Chengdu 610015, P. R. China.

*Correspondence and requests for materials should be addressed to S.G.F. (sgfanglab@zju.edu.cn)

Supplementary Table S1 Gene structure of β-defensins in the giant panda.

Gene	Orientation	Location	Exon1	Exon2	Exon3
Aime-*DEFB132*	-	scaffold3432	10488-10539	9000- 9274	
Aime-*DEFB129*	-	scaffold3432	34574-34631	32410-32867	
Aime-*DEFB128*	+	scaffold3432	78278-78326	79875-80107	
Aime-*DEFB127*	-	scaffold3432	98293-98543	96452-96500	
Aime-*DEFB126*	-	scaffold3432	109555-109612	105562-105770	
Aime-*DEFB126*-like	-	scaffold3432	148464-148521	144226-144476	
Aime-*DEFB125*	+	scaffold3401	25963-26020	41490-41778	
Aime-*DEFB124*	+	scaffold1318	1726347-1726404	1729389-1729539	
Aime-*DEFB123*	-	scaffold1318	1745657-1745714	1739422-1739564	
Aime-*DEFB121*	+	scaffold1318	1768590-1768647	1769840-1769964	
Aime-*DEFB119*	+	scaffold1318	1779016-1779073	1780211-1780404	
Aime-*DEFB117*	-	scaffold1318	1813764-1813821	1808856-1809106	
Aime-*DEFB116*	+	scaffold1318	1850979-1851045	1856001-1856236	
Aime-*DEFB116*-like	+	scaffold1318	1870242-1870299	1880415-1880638	
Aime-*DEFB136*	-	scaffold335	604982- 605036	604360- 604529	
Aime-*DEFB135*	+	scaffold335	652089- 652146	653736- 653902	
Aime-*DEFB134*	-	scaffold335	678157- 678214	675351- 675490	
Aime-*DEFB131*	-	scaffold335	701224- 701281	682822- 682973	
Aime-*DEFB112*	+	scaffold1375	2308954- 2309008	2313892-2314049	
Aime-*DEFB110*	+	scaffold1375	2330805- 2330859	2335741-2335886	
Aime-*DEFB113*	+	scaffold1375	2366478- 2366535	2367249- 2367436	
Aime-*DEFB114*	+	scaffold1375	2374440- 2374497	2391545- 2391687	
Aime-*DEFB108*	+	scaffold2713	13062-13149	17887-18044	
Aime-*DEFB109*	+	scaffold2713	25139-25193	38856-39058	
Aime-*DEFB130*	+	scaffold2713	50401-50458	56470-56648	
Aime-*DEFB107*	-	scaffold2426	27514-27571	26633-26772	
Aime-*DEFB105*	-	scaffold2426	39499-39585	36031-36155	
Aime-*DEFB106*	+	scaffold2426	43832-43880	46476-46624	
Aime-*DEFB_SPAG11B*	+	scaffold2426	54226-54283	55218--55377	59539-59669
Aime-*DEFB_SPAG11*	+	scaffold2426	75632-75686	77109-77290	
Aime-*DEFB103*	+	scaffold2426	96479-96536	97504-97646	
Aime-*DEFB4*	+	scaffold2426	106509-106566	108386-108465	
Aime-*DEFB138*	+	scaffold2426	118993-119050	122020-122147	
Aime-*DEFB139*	-	scaffold2426	129367-129424	127106-127236	
Aime-*DEFB140*	-	scaffold2426	134785-134842	133973-134109	
Aime-*DEFB1*	+	scaffold2426	185019 185178	194476 194621	

Supplementary Table S2 Evolutionary distance between the signal peptide region of each carnivore *DEFB139* and that of non-carnivore *DEFB139* genes.

Gene	dN	dS	dN/dS
Giant panda *DEFB139*	0.35	0.44	0.79
Seal *DEFB139*	0.21	0.50	0.43
Walrus *DEFB139*	0.26	0.46	0.57
Cheetah *DEFB139*	0.29	0.68	0.42
Cat *DEFB139*	0.28	0.69	0.40
Dog *DEFB139*	0.18	0.59	0.31
Polar bear *DEFB139*	0.31	0.44	0.71
Ferret *DEFB139*	0.25	0.41	0.61
Tiger *DEFB139*	0.28	0.72	0.38

Supplementary Table S3 Changes in the selective pressure on different carnivore *DEFB139* genes relative to the pressure on non-carnivore genes.

K is the selection intensity parameter, K>1 indicates that the negative and/or positive selection has intensified, whereas K<1 indicates a relaxed selective pressure. The intensification or relaxation of selection is statistically significant at $p < 0.05$.

Lineage tested	K	LR	P
Giant panda	7.53	3.21	0.07
Polar bear	0.13	0.07	0.79
Cheetah	6.89	0.95	0.33
Dog	0	2.22	0.14
Cat	0	0.53	0.47
Tiger	0	0.53	0.47
Ferret	1.24	0.02	0.90
Walrus	0	0.75	0.39
Seal	0	0.52	0.47

Supplementary Table S4 Physiochemical parameters and Mahalanobis distances of different DEFB139 mature peptides expressed in carnivores.

The isoelectric point and molecular weight were calculated using the Isoelectric Point Calculator, and the instability index was measured with ProtParam. All Cys residues were in a reduced state when values were calculated. A peptide with an instability index below 40 was regarded as stable and with a significantly longer half-life *in vivo*. D_2^2 and D_1^2 represent the Mahalanobis distances calculated with and without the Arg-to-Pro-substituted Aime-DEFB139 mature peptide, respectively. According to the Mahalanobis distances, only Aime-DEFB139 peptide was an outlier and thus indicated in bold.

Peptide	Isoelectric point (pH unit)	Instability index	Molecular weight (KDa)	D_1^2/D_2^2
Aime-DEFB139	9.97	65.80	5.181	**7.53/7.89**
Arg-to-Pro-substituted Aime-DEFB139	9.37	79.75	5.122	NA/0.53
Lewe-DEFB139	8.91	80.93	5.268	3.39/3.83
Odro-DEFB139	8.91	91.57	5.268	3.84/4.40
Acju-DEFB139	9.39	93.99	5.140	1.38/1.52
Feca-DEFB139	9.39	93.99	5.140	1.38/1.52
Calufa-DEFB139	8.76	69.97	5.104	4.21/4.32
Urma-DEFB139	9.15	91.09	5.058	2.90/2.83
Mupufu-DEFB139	8.91	77.86	5.103	2.17/2.37
Pati-DEFB139	9.39	93.99	5.140	1.38/1.52

Supplementary Table S5 ANOVA analyses of differences in antimicrobial activities between the Acm-protected Aime-DEFB139 mature peptide and the Arg-to-Pro-substituted Aime-DEFB139.

$P < 0.05$ indicates a statistically significant antimicrobial activity.

Bacteria	Defensins	Time points	P values
E. coli	Aime-DEFB139	8 h	**0.000**
		18 h	**0.000**
		24 h	**0.000**
	Arg-to-Pro-substituted Aime-DEFB139	8 h	**0.000**
		18 h	**0.000**
		24 h	**0.000**
S. aureus	Aime-DEFB139	8 h	**0.003**
		18 h	**0.000**
		24 h	**0.000**
	Arg-to-Pro-substituted Aime-DEFB139	8 h	0.070
		18 h	**0.003**
		24 h	**0.007**
K. pneumoniae	Aime-DEFB139	8 h	**0.000**
		18 h	**0.000**
		24 h	**0.000**
	Arg-to-Pro-substituted Aime-DEFB139	8 h	**0.000**
		18 h	**0.000**
		24 h	**0.000**
Y. enterocolitica	Aime-DEFB139	8 h	**0.000**
		18 h	**0.000**
		24 h	**0.000**
	Arg-to-Pro-substituted Aime-DEFB139	8 h	**0.000**
		18 h	**0.000**
		24 h	**0.000**

Supplementary Table S6 Student's t-test analyses of differences in antimicrobial activities between the Acm-protected Aime-DEFB139 mature peptide and the Arg-to-Pro-substituted Aime-DEFB139 peptide against *S. aureus*.

P < 0.05 indicates that Aime-DEFB139 had a statistically significantly higher antimicrobial activity than the Arg-to-Pro-substituted Aime-DEFB139.

Time points	Concentrations (mg/L)	P values
18 h	128	**0.000**
	64	**0.001**
	32	**0.001**
24 h	128	**0.000**
	64	**0.000**
	32	**0.000**
	16	**0.000**
	8	**0.000**
	4	**0.002**
	2	**0.003**
	1	**0.006**

Supplementary Table S7 Branch model comparisons of cetacean *DEFB103* and *DEFB140* mature peptide regions.

Model A is a one-ratio model constraining a single ω value to all branches. Model B is a two-ratio model that assigns a dN/dS (ω) to foreground cetacean lineages (ω_1), whereas all remaining lineages share another ω (ω_2). The log likelihood ratio (LR) represents twice the log likelihood difference between the null one-ratio model and alternative two-ratio model. Degrees of freedom (df) indicate the difference between the number of parameters used in the two models. $P < 0.05$ indicates that the alternative model is significantly more fit than the null model. Model B is more fit than model A, suggesting that both cetacean *DEFB103* and *DEFB140* have experienced a significantly increased positive selection compared with the selective pressure on other mammals.

Model comparisons	LR	df	ω_2/ω_1	p
103A *vs.* 103B	22.48	1	0.27/1.41	**0.00**
140A *vs.* 140B	7.83	1	0.16/0.75	**0.01**

Supplementary Table S8 Primers used to fill the gaps smaller than 5000 bp in the giant panda defensin cluster.

To enhance amplification and ensure primer specificity, nested PCR was used and BLAST alignment was used to assess the primers against the giant panda genome using Primer-BLAST during their design. The corresponding PCR conditions are detailed in the Supplementary Note.

Primer name	Primer sequence (5´ to 3´)	Size (bp)	Ta (°C)
103-SPAG11 gap_N1F	ATGAACGGTAACAGTGTTGGTGG	1465	59
103-SPAG11 gap_N1R	CAGCTCAGTTGTGAGGCAGAATAA		
103-SPAG11 gap_N2F	TGTGGACGTGATGAGGCTATG	507	59
103-SPAG11 gap_N2F	ACTTGAGAACGGTCCCGATGC		
109-130 gap_N1F	AGTCTGTTGATAGGCTGAAAT	995	54
109-130 gap_N1R	CATAAAAGGAAAGTAGTGTAGG		
109-130 gap_N2F	TTTTATAAGTGACACTTGCATTC	472	53
109-130 gap_N2R	ATAGAATAGTCTGGTGATGGGTA		
116-117 gap1_N1F	TCATCAGTTATCCCAAGCAAG	1425	55
116-117 gap1_N1R	TGAGCAGTGTCATAGGAGGTT		
116-117 gap1_N2F	TGGGAAATAAAAACACACTCACGAT	1197	57
116-117 gap1_N2R	ACTAAGAAACCCAAGAACCCTC		
116-117 gap2_N1F	CTGAGGATGCAGGCTATT	2268	54
116-117 gap2_N1R	CTGGAGTCTTGCGAACC		
116-117 gap2_N2F	TGATCTGAGGGATCTGGGACTG	780	56
116-117 gap2_N2R	TCATGGATGAGGGCAAGAAAAT		
116like-116gap1_N1F	GCAATGAGTACAGGCTAACG	1854	54
116like-116gap1_N1R	TACAGGCAAAGAAAGCAAGG		
116like-116gap1_N2F	TTTTACTATGCTTTTAGGTTTC	639	49
116like-116gap1_N2R	AATATCTTTCTAGCCTTGTT		
116like-116gap2_N1F	GATGGCATTCAAAGGTGAC	4937	57
116like-116gap2_N1R	ACTGACTGAGCGAAAGGAG		
116like-116gap2_N2F	CTCCCCCAGGTTATTGAGATATGAC	559	60
116like-116gap2_N2R	ACTGAACTCAAAGAAACAGAGTGGTAG		
116like-116gap2_N1F	GATGGCATTCAAAGGTGAC	4937	57
116like-116gap2_N1R	ACTGACTGAGCGAAAGGAG		
116like-116gap3_N2F	ATAACCATTTTTCGTGCATGAAGTG	470	56
116like-116gap3_N2R	TGATCAACAGGTGCATGAATAGATG		
116like-116gap2_N1F	GATGGCATTCAAAGGTGAC	4937	57
116like-116gap2_N1R	ACTGACTGAGCGAAAGGAG		
116like-116gap4_N2F	ATTCATGCACCTGTTGATCATTTGTATG	768	60
116like-116gap4_N2R	TAGCACCAGAAACCATAAGACATCTCTG		
126like-126gap1_N1F	TGTTACCTGTTTCCTGGTTCAT	2986	55
126like-126gap1_N1R	ATTGGCTAATATCTTCCTTGTTCTC		
126like-126gap1_N2F	GGCTTAGTTTCTATGCAAGGAT	1046	54
126like-126gap1_N2R	AAATAATGGCTCTGATATGACC		

126like-126gap1_N1F	TGTTACCTGTTTCCTGGTTCAT	2986	55
126like-126gap1_N1R	ATTGGCTAATATCTTCCTTGTTCTC		
126like-126gap2_N2F	AAGTCAATAAATGGCGACAAAT	1055	53
126like-126gap2_N2R	CTAACCAGGCACCTAACTAATC		
126like-126gap3_N1F	TTACACTTCCTTTCCAGCCACTCC	2446	60
126like-126gap3_ N1R	CTTTGCTTCGTACAGCACCAACCT		
126like-126gap3_ N2F	GAATGAAAGCACATATCAATAAAGG	1178	54
126like-126gap3_ N2R	AAGGAGAATAATGAGAAGCCAAA		
129-128gap_N1F	CAGGGCTTGATCTTACCACC	1306	58
129-128gap_N1R	AACCCATTACAGTCATGCTTCC		
129-128gap_N2F	GATGTGGGTATGCTAGTGATG	868	53
129-128gap_N2R	ATTTGGTGGCTTGGATTT		

Supplementary Table S9 Primers used to amplify large gaps or pick BAC clones covering large gaps, and the universal primers used to amplify big gaps or BAC clone templates. BLAST was used to align all primers against the giant panda genome with Primer-BLAST to ensure primer specificity. For detailed PCR conditions, please refer to the Supplementary Note.

Primer name	Primer sequence (5´ to 3´)	Size (bp)	Ta (℃)
126like-126gap4_F	CATCATCAACTCTAACACGA	7597	56
126like-126gap4_R	GACCTTAATAATAGCCCATC		
126universal_F	GRARGAGTYCYAACATCTCTTCA	1240	58
126 universal _R	GCCTCAYTTGTGYCTGGCMTCT		
126like-116like BAC_F	CCACGGTCAACATTCATA	304	52
126like-116like BAC_R	AGAGGTTCTTGGAGGATA		
126like universal _F	YCCASKTGGTYTCAGGTAAA	158	57
126like universal _R	TRTTCTGAAGAARTARRGCAAA		
116like universal _F	GTCAGTYATGAAGCCCTRTTTAAT	101	57
116like universal _R	CARRACCCCAGAAYYTTTGA		
129-128gap2_F	TCCTTCTGGGAATACAACCT	5958	63
129-128gap2_R	TACCAGCACATCCTGAACAC		
128 universal _F	CTGCCTYRGGAACTAATCT	320	50
128 universal _R	TTTARGARGAGTGGASACC		
130BAC_F	CAACGAGATACCACCTCATACCAGA	867	60
130BAC_R	AAACATTCTAAACGTGGCACCTATT		
130 universal _F	TYCCNGGRCARAARCAGT	150	53
130 universal _R	TGGGARCNGGHGTTGSRTA		

Supplementary Table S10 Primers used to examine the expression pattern of different β-defensins.

Thirty-six primer pairs were designed, but only those that detected expression are listed. The host *GAPDH* gene (glyceraldehyde-3-phosphate dehydrogenase) was used as an internal reference. For detailed PCR conditions, please refer to the Supplementary Note.

Primer name	Primer sequence (5´ to 3´)	Size (bp)	Ta (℃)
Aime1_F	TGCCTACACCGGGCATCC	120	60
Aime1_R	TGACCTCTGGCCGAAACCTG		
Aime103_F	GAGGATCTATTACCTTCTCCTCCTG	198	61
Aime103_R	TTCTTTCTTCGGCAGCACTT		
Aime139_F	GTCCTCTTCATCTCTCACACAAT	110	60
Aime139_R	TGCGAACCAGACAGTTTCTC		
Aime140_F	ATGGTCCTGCAGGTACTGC	111	57
Aime140_R	GGAAGGGCTCACAGTTTGGT		
GAPDH_F	GGGGTGATGCTGGTGCTGAGTATGT	264	62
GAPDH_R	TGAGTCCCTCCACGATGCCGAAGT		

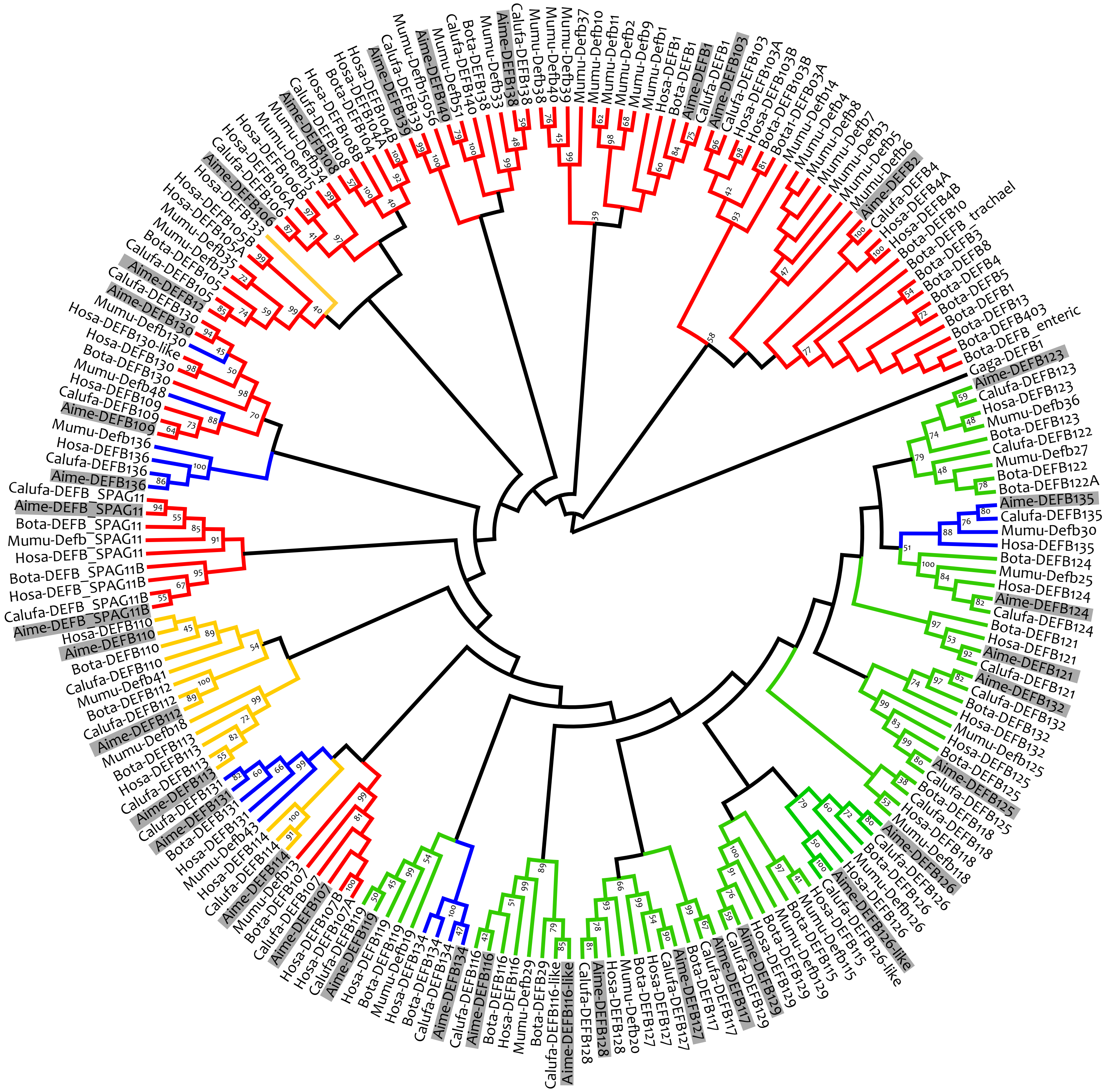

Supplementary Figure S1 Detailed neighbour-joining tree of β-defensins expressed in the giant panda, dog, cattle, mouse, and humans. The names of the genes start with the abbreviation of the Latin species name followed by the name of the β-defensins. The chicken β-defensin 1 (Gaga-DEFB1) was used as an outgroup, and bootstrap values under 40 are not shown. Lineages from the same chromosome are depicted in the same colour, and orthologs in the giant panda are shaded in grey.

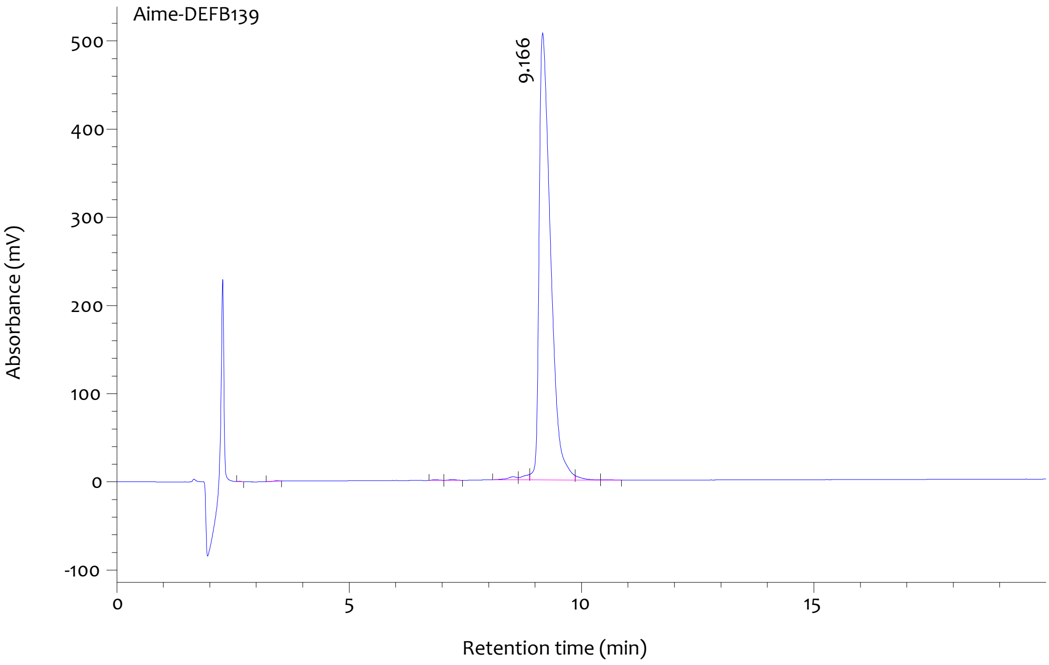

a

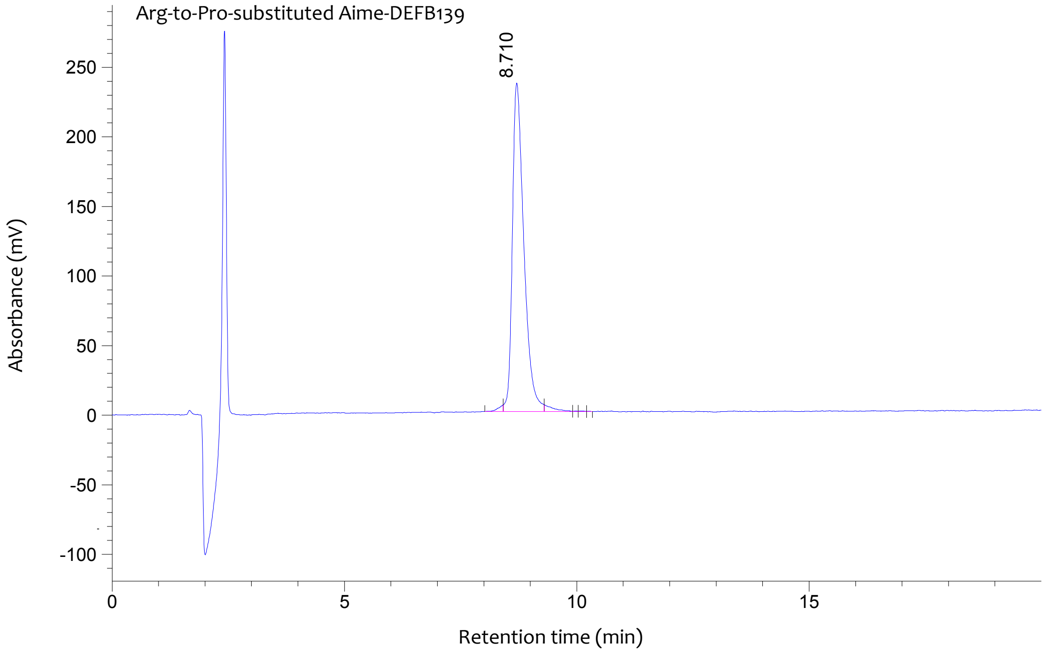

b

Supplementary Figure S2 HPLC chromatograms of the synthesized Aime-DEFB139 and Arg-to-Pro-substituted Aime-DEFB139 with their Cys residues protected by Acm. **a**) Acm-protected Aime-DEFB139. **b**) Acm-protected Arg-to-Pro-substituted Aime-DEFB139.

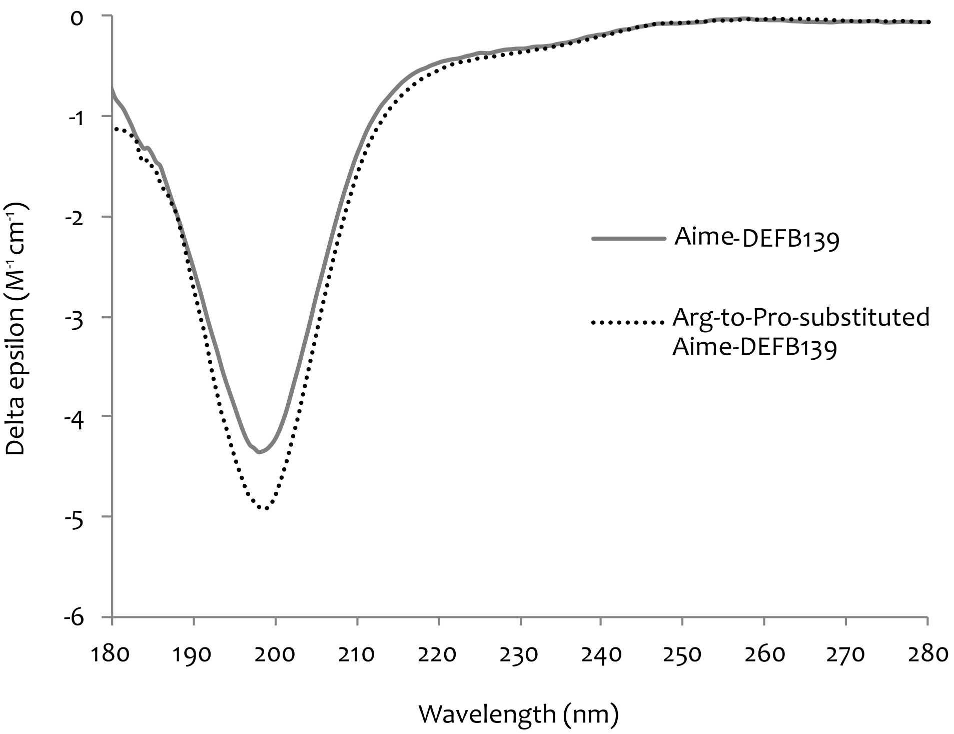

Supplementary Figure S3 CD spectra of Acm-protected Aime-DEFB139 and Arg-to-Pro-substituted Aime-DEFB139.

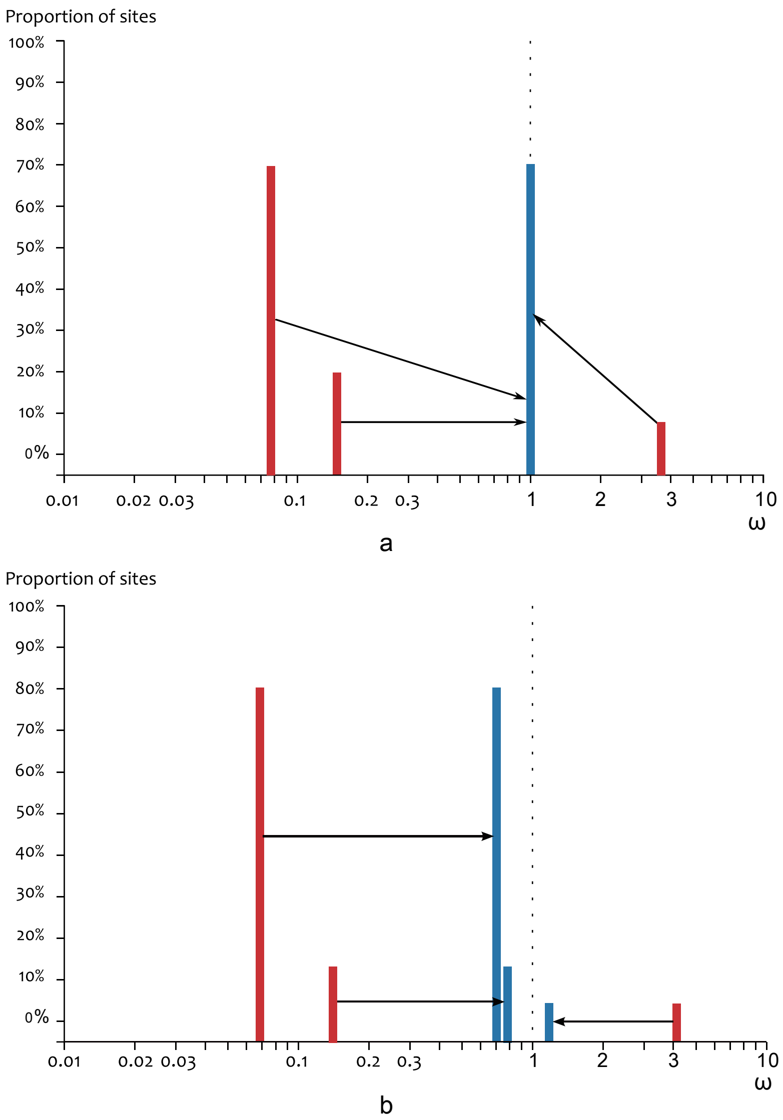

Supplementary Figure S4 The relaxed selective pressures on the mature peptide region of cetacean *DEFB103* (a) and *DEFB140* (b). Blue bars represent cetacean lineages, red bars represent all remaining mammals, and lines with arrows indicate the direction of the changes in ω. **a**) In other mammals, approximately 90% of the sites are under negative selection and 10% of sites have ω > 1. However, in cetaceans the proportion of sites under negative selection has decreased, whereas additional sites, such as those that experienced positive selection in other mammals, have become selectively neutral. **b**) Compared with the selective pressures on other mammals, those on sites in cetaceans have changed towards neutral selection. The results suggest that the increased selective pressure revealed by branch model comparisons (Supplementary Table S6) is actually a relaxation of both purifying and positive selection.

Supplementary Methods

Detecting selective pressure on the carnivore DEFB139 signal peptide

The best nucleotide substitution model for signal peptide regions was estimated using MEGA 6.0 to acquire the transition/transversion bias (R). After defining the "Carnivora" and "non-Carnivora" groups, the average rates of synonymous (dS) and nonsynonymous (dN) substitutions in the signal peptide region of *DEFB139* genes and the significance of the selection was evaluated by a Z-test of selection in MEGA 6.0 using a modified Nei and Gojobori method with the Jukes-Cantor correction, the estimated R, 1,000 bootstrap replications, and a 90% site coverage cut-off. The evolutionary distance (represented by dN/dS) between the signal peptide of each carnivore *DEFB139* gene and non-carnivore *DEFB139* gene was calculated using the "between group mean distance" analysis in MEGA 6.0 with the settings listed above.

RELAX analysis of changes in the selective pressure on carnivore β-defensin genes

The RELAX program calculates changes of the foreground branch relative to the background branch in proportion of sites with different ω categories to test the significance of alterations in selective pressure. Each time a different carnivore lineage was set as the foreground branch to test it against all non-carnivores to determine whether the intensification of selective pressure on the panda defensins was more significant. To determine the influence of transformation of lifestyle and living environment on the evolutionary scheme of β-defensins, we also conducted branch modelling and RELAX analyses on the cetacean *DEFB1*, *DEFB103*, *DEFB139*, and *DEFB140* using the entire coding sequence and mature peptide region. The procedure was the same as in the analysis of panda defensins, except that the entire cetacean lineage was used as the foreground branch. The analysis revealed that the cetacean *DEFB103* and *DEFB140* genes were under a relaxed selective pressure regardless of whether the entire sequence or mature peptide region was evaluated. Only the results derived from the mature peptide region are presented.

Supplementary Note

Confirmation of Aime-*DEFB139* as a unique gene in the giant panda

To confirm that the Pro-to-Arg mutation is a unique substitution rather than a polymorphism in the giant panda, we amplified the mature peptide region containing the mutation site in 49 individuals from different populations, including 28 individuals from a Sichuan nominal subspecies (*A. m. melanoleuca*) and 21 individuals from the Qingling subspecies (*A. m. qinlingensis*) [1]. All individuals presented monomorphisms at the amplified region; thus, it is likely that the mutation is unique.

The amplification was performed using preserved DNA from a previous study with the following primers: 139mature_1 forward (5'-GCCTCCACATAACACCATAA-3') and 139mature_2 reverse (5'-ATTGAGCGTGACTTCTTCG-3'). The amplified product had a size of 430 bp, and the annealing temperature used in the PCR method was 55 °C. The DNA from some skin samples had degraded; therefore, a second round of amplification was performed using the following primers: 139mature_2 forward (5'-GTGAAGTGTACCATTTCTGTGACTC-3') and 139mature_2 reverse (5-'AAGCTCCTTCCAGGGCAT-3'). The product size was 100 bp, and the annealing

temperature used was 53 °C. The PCR conditions are listed below.

PCR conditions used in the experiment

Conditions for nested PCR:

The first round:

Initial denaturation	94 °C	5 min	
Denaturation	94 °C	30 s	22 cycles
Annealing	Ta °C	30 s	
Extension	72 °C	30 s/kb	
Final extension	72 °C	7 min	

The second round:

Initial denaturation	94 °C	5 min	
Denaturation	94 °C	30 s	31 cycles
Annealing	Ta °C	30 s	
Extension	72 °C	30 s/kb	
Final extension	72 °C	5 min	

The enzyme used for filling big gaps (>5 kb) was the Q5 high-fidelity DNA polymerase. The PCR conditions were as follows:

Preheat	98 °C		
Initial denaturation	98 °C	3 min	
Denaturation	98 °C	10 s	33 cycles
Annealing	Ta °C	30 s	
Extension	72 °C	45 s/kb	
Final extension	72 °C	2 min	

The following conditions were used for all other PCR:

Initial denaturation	94 °C	5 min	
Denaturation	94 °C	30 s	32 cycles
Annealing	Ta °C	30 s	
Extension	72 °C	30 s/kb	
Final extension	72 °C	5 min	

Supplementary Data S1 Sequence alignments for all β-defensins in humans, mice, cattle, dogs, and pandas.

Exons were translated and then aligned separately. Dashes indicate the gaps, and three gaps were used to separate the signal sequences and mature sequences. Data are provided in fasta format.

Supplementary Data S2 All β-defensin genes in humans, mice, cattle, dogs, and pandas.

Scaffold and coordinate information are included. Data are provided in fasta format.

Supplementary Data S3 Sequence alignments of DEFB1, DEFB103, DEFB139, and DEFB140 (in fasta format).

Two exons were translated and then aligned separately. Dashes indicate gaps, and three gaps were used to separate the signal sequences and mature sequences. Data provided in fasta format.

Supplementary Data S4 Orthologs of *DEFB1*, *DEFB103*, *DEFB139*, and *DEFB140* genes. Coordinate information are included. Data are provided in fasta format.

Reference

1. Chen, Y.Y. *et al.* Natural selection coupled with intragenic recombination shapes diversity patterns in the major histocompatibility complex class II genes of the giant panda. *J. Exp. Zool. (Mol. Dev. Ecol.)* **314B,** 208–223 (2010).
